# Lemon Extract Reduces Angiotensin Converting Enzyme (ACE) Expression and Activity and Increases Insulin Sensitivity and Lipolysis in Mouse Adipocytes

**DOI:** 10.3390/nu12082348

**Published:** 2020-08-06

**Authors:** Shilpa Tejpal, Alan M. Wemyss, Claire C. Bastie, Judith Klein-Seetharaman

**Affiliations:** 1 Division of Biomedical Sciences, Warwick Medical School, University of Warwick, Gibbet Hill, Coventry CV4 7AL, UK; tejpalshilpa@gmail.com; 2 Department of Chemistry, University of Warwick, Gibbet Hill, Coventry CV4 7AL, UK; A.Wemyss@warwick.ac.uk; 3 Department of Chemistry, Colorado School of Mines, Golden, CO 80401, USA

**Keywords:** 3T3-L1 adipocytes, lipolysis, angiotensin converting enzyme (ACE) activity

## Abstract

Obesity is associated with insulin resistance and cardiovascular complications. In this paper, we examine the possible beneficial role of lemon juice in dieting. Lemon extract (LE) has been proposed to improve serum insulin levels and decrease angiotensin converting enzyme (ACE) activity in mouse models. ACE is also a biomarker for sustained weight loss and ACE inhibitors improve insulin sensitivity in humans. Here, we show that LE impacts adipose tissue metabolism directly. In 3T3-L1 differentiated adipocyte cells, LE improved insulin sensitivity as evidenced by a 3.74 ± 0.54-fold increase in both pAKT and GLUT4 levels. LE also induced lipolysis as demonstrated by a 16.6 ± 1.2 fold-change in pHSL protein expression levels. ACE gene expression increased 12.0 ± 0.1 fold during differentiation of 3T3-L1 cells in the absence of LE, and treatment with LE decreased ACE gene expression by 80.1 ± 0.5% and protein expression by 55 ± 0.37%. We conclude that LE’s reduction of ACE expression causes increased insulin sensitivity and breakdown of lipids in adipocytes.

## 1. Introduction

More than 2 billion people are classified as overweight or obese worldwide [1]. There are many approaches available to lose weight from surgical or drug-based interventions to different diet programs. The surgeries involve gastric bypass or gastrectomy, leading to massive weight loss in patients. However, patients need to be on a lifelong controlled diet and regular exercise. It comes with risks such as internal bleeding, pulmonary embolism and death [2,3]. Weight loss drugs, on the other hand, work by suppressing appetite or increasing metabolism [4]. However, these drugs are associated with side effects such as myocardial infarction and stroke [5]. The most common treatment of obesity involves dietary plans such as the Atkins Diet, the Mediterranean Diet, or the Keto-Diet, to name just a few. Generally, different diets fall into categories, such as low-carbohydrate diets, low-fat diets, caloric restriction, intermittent fasting, and alternate fasting. Many of these dietary interventions have been subjected to clinical trials to evaluate their effectiveness in promoting weight loss and improving markers of metabolic health, such as insulin sensitivity. For example, combining low-carbohydrate with high-protein intake has been shown to maintain low insulin levels and reduce the ratio between low-density lipoprotein (LDL) to high-density lipoprotein (HDL) [6,7]. However, the consumption of more calories originating from fat, especially saturated fatty acids, can potentially increase the risk of developing coronary heart disease [8]. Intermittent fasting can also increase insulin sensitivity and has been shown to protect against oxidative damage of proteins, lipids, and DNA [9]. Both, intermittent fasting and caloric restriction change serum insulin-like growth factor-1 and cortisol levels and improve immune function [9,10]. A type of calorie-restricted diet known as very low-calorie diets (VLCD) involve consumption of <500 Kcal per day and are hard to maintain [11]. They can cause mineral and vitamin loss during the early intervention period [12]. Individuals following very-low-calorie diets often experience headache, dizziness, fatigue, gastrointestinal problems, and nausea [13,14].

Lemon dieting is a VLCD diet in use since the 1940s, which provides 800 to 1000 kcal per day [15]. The program often consists of Neera syrup (a blend of maple and palm tree syrups) and lemon juice. The syrup is designed to have high amounts of minerals and trace elements. Therefore, the lemon detox program can provide higher minerals and vitamins than other VLCDs [15]. Instead of syrup, sugar can also be used, without the benefits of minerals, because the active weight loss ingredient is the lemon juice. However, the mechanisms by which lemon juice assists weight loss are not clear. Lemon juice is a known diuretic, and thus the mechanism may involve the angiotensin-renin system (RAS).

Benefits of plant bioactive compounds on metabolism have become a focus of multidisciplinary studies, and several plant extracts have been identified for the prevention of obesity [16]. Based on their nutritional value, such extracts may have physiological benefits and reduce the risk of chronic diseases [17,18]. For example, citrus fruit species are associated with decreased inflammation and reduced oxidative stress markers [19]. Other studies have found bergamot, grapefruit and orange juice to be associated with decreased total cholesterol, LDL and glucose concentration in humans [20,21,22]. Similar results have also been found in rats after lemon juice administration [23].

Obesity is associated with numerous co-morbidities such as type-2 diabetes, cardiovascular disease (CVD), metabolic syndrome, hypertension and several cancers [24,25,26,27]. The cellular mechanisms that are known to majorly contribute to these associations are related to insulin resistance and glucose uptake, lipolysis and regulation of blood pressure through RAS. Insulin resistance is defined as impaired sensitivity to insulin (normal or elevated), which mediates glucose disposal [28]. Expression levels of the glucose transporter type 4 (GLUT4), responsible for insulin-mediated glucose uptake [29], has been reported to be reduced in both, rodents and human with insulin resistance [30,31]. Even when GLUT4 is eliminated, specifically in adipose tissue, the absence of GLUT4 leads to a whole-body disturbance in glucose homeostasis, resulting in insulin resistance even in skeletal muscle and liver [32].

Maturation of adipocytes lead to increased expression of peroxisome proliferator–activated receptor γ (PPARγ), fatty acid binding protein (FABP4), lipoprotein lipase (LPL), and adiponectin [33,34]. This results in an increased accumulation of triacylglyceride (TAG). Lipolysis is the breakdown of stored TAGs in adipocytes into glycerol and fatty acid (FA) [35]. The breakdown is regulated by different enzymes acting in an orderly fashion. Perilipin is one such enzyme and is located on the surface of lipid droplets (LD) in adipocytes. Its phosphorylation by protein kinase A (PKA) via cyclic adenosine monophosphate (cAMP) pathway leads to di- and triglyceride breakdown [36]. Hormone sensitive lipase (HSL), following PKA stimulation, translocates from the cytosol to LD surfaces leading to lipolysis [37]. Adipose triglyceride lipase (ATGL), TAG hydrolases and adiponectin are some other proteins involved in lipolysis.

The link between RAS and obesity has been known since 1968 [38]. It is, therefore, perhaps not surprising that profiling of blood proteins and steroid hormones revealed that angiotensin converting enzyme (ACE) concentration is a predictor of weight maintenance [39]. ACE is a zinc metallopeptidase involved in hydrolysis of angiotensin (ang) along with breakdown of bradykinin into inactive products [39,40,41]. ACE activity is a critical component of RAS responsible for the conversion of ang I to ang II [42]. Ang II is involved in decreased insulin sensitivity, increased reactive oxygen species generation, decreased glucose uptake, regulation of blood pressure and electrolyte balance [43]. Thus, ACE plays a key role in regulating blood pressure and electrolyte balance [44]. ACE inhibitors are widely used in the treatment and prevention of hypertension [45]. Increased production of ang II is associated with increased lipogenesis in human adipose cells [46]. It could induce differentiation leading to formation of mature adipocyte [47].

To better understand the mechanism of the beneficial effects of lemon juice on weight loss, the current study focuses on the effects of lemon extract (LE) on ACE expression, insulin sensitivity and fat accumulation in 3T3-L1 adipocytes.

## 2. Materials and Methods

### 2.1. Reagents

Dulbecco’s modified Eagle’s medium (DMEM), fetal bovine serum (FBS), new calf serum (NCS), Ham’s F12, antibiotics, and other cell culture products were from Invitrogen and Thermo Fisher Scientific, Oxford, UK. AKT, pAKT, GLUT4, GADPH, perilipin, HSL and pHSL antibodies were from Cell Signaling Technology, London, UK. ACE 1 antibody was from Abcam Plc., Cambridge, UK. Polyvinylidene difluoride (PVDF) membranes used for Western blots were from Thermo Fisher Scientific, Oxford, UK. TriZol was from Invitrogen while Taqman gene expression assay kit for quantitative PCR and high capacity RNA to cDNA kit were from Applied Biosystems, Warrington, UK.

### 2.2. Cell Culture

3T3-L1 cells were maintained in DMEM/F12 medium supplemented with 10% NCS and Penicillin-Streptomycin (Pen-Strep) antibiotic solution. They were differentiated as in [48]. Briefly, cells were plated and cultured to confluency (Day 0). They were then left for two days at confluence prior to addition of the differentiation medium, i.e., DMEM/F12 medium supplemented with 10% FBS, insulin, 3-isobutyl-1-methylxanthine (IBMX), dexamethasone and Pen-Strep antibiotic solution (Day 2). They were then treated for 48 h (until Day 4) with the differentiated medium. Following this, they were treated with maintenance medium (DMEM/F12 supplemented with 10% FBS and Pen-Strep solution) for 48 h (until Day 8).

### 2.3. Preparation of Lemon Extract (LE)

One lemon was squeezed into a 50 mL falcon tube. This solution was then frozen by placing the 50 mL falcon tube at −20 °C for 24 h. The frozen solution was kept on dry ice for 2 h before freeze-drying. The solution was lyophilised overnight in an instrument named Alpha 2–4 LD plus made by Martin Christ Gefriertrocknungsanlagen GmbH (Osterode am Harz, Germany). The lyophilised powder was re-suspended in 10 mL of DMEM/F12. The pH was adjusted to 7.0 by adding sodium hydroxide.

### 2.4. RNA Isolation and Gene Expression

RNA was prepared using TriZol reagent. Real-time quantitative PCR was performed using Taqman expression assay for ACE as described by the supplier. Briefly, complementary DNA (cDNA) was synthesized from 100 ng of total RNA with high capacity RNA to cDNA kit. Quantitative PCR was performed in Applied Biosystems 7500 Fast Real-Time PCR System instrument in the presence of Taqman gene expression assay and master mix. All experiments were done in 3 biological and experimental repeats.

### 2.5. Protein Extraction

Protein was extracted using radio immune precipitation (RIPA) lysis buffer complemented with a Protease inhibitor cocktail (Set V, Calbiochem, Dorset, UK) and a phosphatase inhibitor cocktail (P5726 (Sigma, Dorset, UK)). Cell lysates were vortexed every 2 min and kept on ice for 10 min before being centrifuged at 12,000 rpm for 10 min at 4 °C. Supernatants were collected and total proteins were quantified using a Modified Lowry assay (Thermo Scientific, Oxford, UK). All experiments were done in 3 biological and experimental repeats.

### 2.6. Western Blot Analysis

Proteins (30 μg) were separated on a 10% resolving and 4% stacking polyacrylamide-gel and electroblotted onto PVDF membranes. Blots were blocked with a 3% BSA in Tris-buffered saline and 0.1% Tween-20 (TBST) for 60 min at room temperature and then incubated overnight at 4 °C with antibodies against pHSL (1:1000), ACE 1 (1:200), p (S473) AKT (1:1000), GLUT4 (1:1000) and GAPDH (1:1000) in TBST containing 1% BSA. Blots were washed 3 times for 15 min with TBST and incubated with appropriate horse-radish peroxidase-conjugated secondary antibodies (1:10,000 in TBST) for 60 min at room temperature. Membranes were washed 3 times for 15 min in TBST and antigen–antibody complexes were visualized by chemiluminescence using an ECL kit (Thermo Scientific Fisher, Oxford, UK).

### 2.7. ACE Inhibitory Activity Assay

ACE inhibitory activity was measured by a fluorometric assay following the method of [49]. A potential ACE inhibitor would prevent conversion of Abz-GLY-PHe(NO2)-Pro to Abz-GLY by inhibiting ACE enzyme. A total of 50 µL of LE (0, 50, 100 and 500 µg/mL) followed by 50 µL ACE (3 mU/mL) solution was added in each well of a black 96-well plate. The mixture was incubated at 37 °C for 10 min. A quantity of 200 µL of Abz-GLY-PHe(NO2)-Pro in 150 mM Tris base (pH 8.3) with 1.125 M NaCl was added to each well and fluorescence was measured at 360 nm (excitation) and 430 nm (emission) for t = 0. The plate was then incubated for 30 min and reading was taken as mentioned above. %inhibitory activity was calculated following the method in [50].

### 2.8. Free Glycerol Release Assay

Differentiated 3T3L1 adipocytes were pre-incubated with DMEM/F12 (without phenol red) for 2 h before being treated with LE or phosphate buffer saline (PBS) for 0, 2, 4, 6 and 24 h. A 200 µL aliquot was taken at t = 0, 2, 4 6, and 24 h. After t = 24 h, cells were lysed for protein extraction followed by protein quantification. Free glycerol amount was quantified using a free glycerol kit (Sigma, Dorset, UK). The amount of free glycerol was normalized by the amount of protein.

### 2.9. Insulin Sensitivity Assay

Cells were differentiated on a 12-well plate and starved overnight (Day 7) by addition of DMEM/F12 supplemented with Pen/Strep (FBS deprived) to the wells. On Day 8, they were incubated with 100 µg/mL of LE for 10 h or PBS (control) and then treated with 100 nM of insulin for 15 min or PBS (negative control).

### 2.10. Cell Viability Assay

The cells were trypsinized as above and 50 µL of cell suspension was added in an eppendorf tube. Equal parts of 0.4% trypan blue dye were added to the cell suspension and mixed by pipetting up and down. Place the cover slip on the hemocytometer and 10–20 µL of cell suspension on one side of the hemocytometer. The hemocytometer was placed on the stage of a light microscope and the cells were counted in each large corner. The percentage of viable cells were calculated by dividing the number of viable cells by the number of total cells and multiplying by 100.

### 2.11. Oil Red O Staining

Cells were washed twice with PBS before being fixed in 10% formalin (Sigma-Aldrich, Dorset, UK) for 60 min. Cells were then washed with water and incubated with 60% isopropanol in water for 5 min. Filtered Oil Red O solution (3 parts 3 mg/mL Oil Red O powder and 2 parts water) was added to the cells for 20 min at room temperature. The cells were then washed with water up to 5 times and covered with water to view under the microscope. For quantification, the water was removed and allowed to dry. The dye was eluted in 100% isopropanol and incubated for 10 min with gentle shaking. The isopropanol was pipetted up and down several times ensuring that the dye was in the solution. Absorbance was taken at 500 nm with 100% isopropanol as a blank.

### 2.12. High-Performance Liquid Chromatography (HPLC)

Agilent 1260 Infinity HPLC (Agilent Technologies, Stockport, UK) was used for analysis. LE was separated on a C-18 column (250 mm × 5 mm × 4.6 mm). The mobile phase consisted of 0.04% formic acid in water (A) and acetonitrile (ACN) in 0.04% formic acid (B). An injection volume of 50 µL was used with the gradient conditions given in Table 1. The column temperature was set at 25 °C and the sample absorbance at 278 nm was monitored throughout each run.

### 2.13. Mass Spectroscopy

Following the optimisation of the HPLC conditions, different compounds in LE were identified using HPLC-MS. Data were acquired on the MaXis II Q-TOF instrument (Bruker AXS, Coventry, UK) coupled with Dionex 3000RS UHPLC and the column used was Agilent Zorbax C18, 100 × 2.1 mm (Agilent Technologies, Stockport, UK). The mobile phase used was water (A) and acetonitrile (B) with 0.1% ammonium or formic acid, for measurements in positive or negative mode, respectively. The same gradient, as in HPLC, with a reduced flow rate of 0.2 mL/min was used.

### 2.14. Statistics

Results are expressed as mean ± S.D. Differences between cells or treatments were tested for statistical significance using the unpaired Student’s t test.

## 3. Results

### 3.1. Lemon Extract Decreases ACE Gene and Protein Expression as Well as ACE Enzymatic Activity

**Figure 1:**
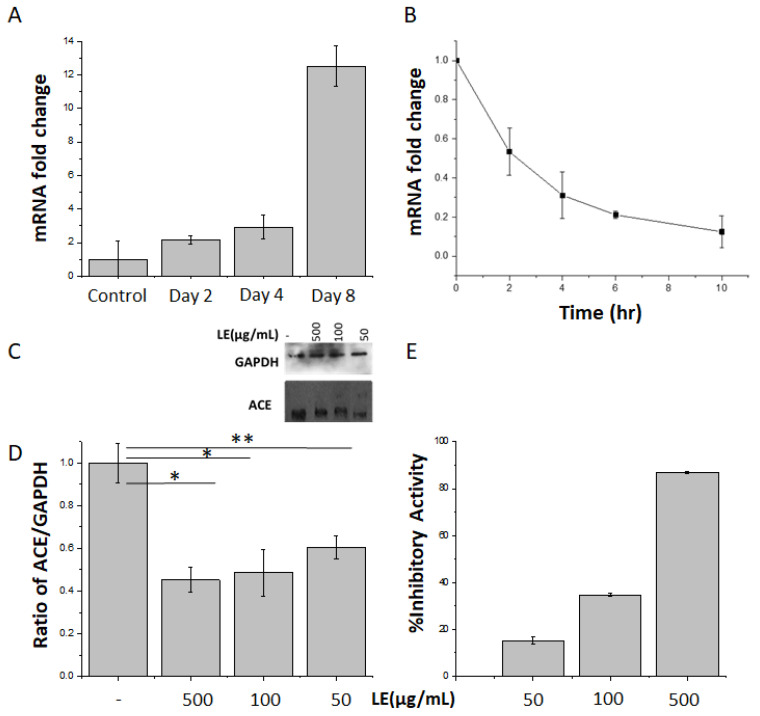
Total lemon extracts (LE) decrease angiotensin converting enzyme (ACE) expression and activity. (**A**) ACE mRNA expression levels were quantified at the indicated days during 3T3L1 adipocyte differentiation. *n* = 3 independent experiments, *p* < 0.001. (**B**) Fully differentiated 3T3L1 adipocytes were treated or not with LE (100 µg/mL) for the indicated times and ACE mRNA expression levels were quantified. *n* = 3 experiments, *p* < 0.05. (**C**) 3T3L1 differentiated adipocytes were incubated with 50, 100 or 500 µg/mL of LE for 10 h on day 8. ACE and GAPDH protein expression were assessed by immunoblotting. GAPDH was used as internal control. n =3. (**D**) Signal quantification of ACE and GAPDH immunoblots (**E**) LE inhibitory effects on ACE activity. Cells were treated with LE (50, 100 and 500 µg/mL) and conversion of Abz-GLY-PHe(NO2)-Pro to Abz-GLY was quantified as described in methods. *n* = 3. ** significant at *p* = 0.01 and * significant at *p* = 0.05.

A significant (*p* < 0.002) and robust (12.00 ± 0.05-fold) increase in ACE gene expression was observed during 3T3L1 adipogenesis (Figure 1A). Incubation of mature differentiated 3T3L1 adipocytes with total LE (100 µg/mL) resulted in a significant reduction in ACE gene expression, which was reduced by 80.0% ± 0.5% after 10 h (Figure 1B). Differentiated 3T3-L1 cells were treated with LE doses of 50, 100 and 500 µg/mL. There was no significant change in ACE protein expression within the 3 doses (Figure 1, and for individual repeats see Figure S1 and Table S1) used, likely because 50 µg was already the maximum effect on the protein expression (Figure 1C). However, there was a 40.0–55.0 ± 0.4% (*p* = 0.01) reduction in protein expression compared with untreated cells (Figure 1D). This was tightly correlated with ACE reduced activity (15% up to 87%) with increasing concentrations of LE (Figure 1E). ACE activity was quantified by measuring the conversion of ACE substrate Abz-GLY-Phe(NO2)-Pro into Abz-GLY product. Consistent with previous studies [23], it was observed that LE prevented this reaction, demonstrating inhibition of ACE activity.

### 3.2. Lemon Extract Decreases Lipid Droplet Numbers in 3T3-L1 Adipocytes

**Figure 2:**
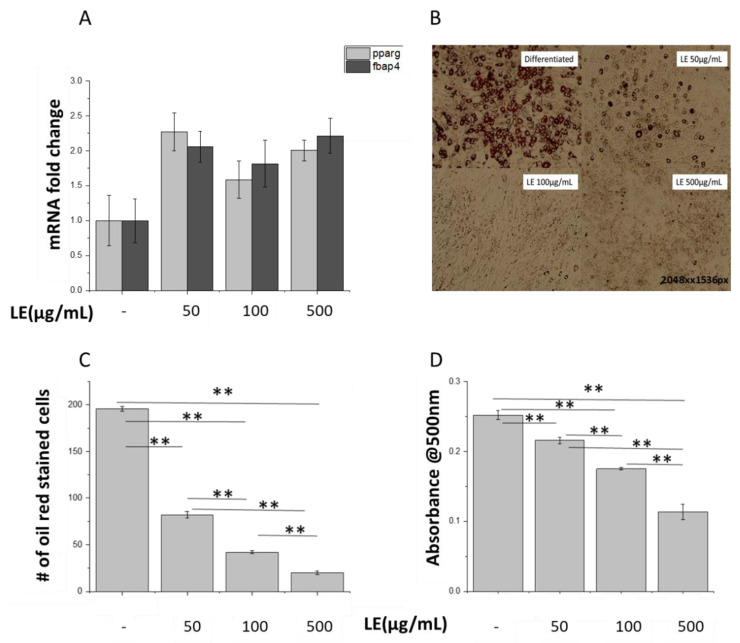
Lemon extract (LE) decreases lipid droplets in 3T3-L1 adipocytes. (**A**) PPARγ and FABP4 mRNA expression levels were quantified in adipocytes treated with and without LE (100 µg/mL) for 8 days. *n* = 3. (**B**) Lipids were visualized using Oil Red staining. Images are representative of 3 experiments. (**C**) Staining was quantified using ImageJ by calculating number of oil red stained cells in the section shown in (**B**), ** significant at *p* = 0.01. (**D**) Quantified oil red stain in treated and untreated adipocytes, ** significant at *p* = 0.01.

Peroxisome proliferator-activated receptor gamma (PPARγ) and Fatty Acid-Binding Protein 4 (FAPB4) are known markers of late adipose differentiation [33]. Differentiated mature adipocytes treated with LE showed similar PPARγ and FABP4 gene expression compared to control untreated adipocytes, suggesting that LE treatment was not affecting the adipogenesis process (Figure 2A). However, visualization of adipocytes by Oil red O staining showed that LE-treated adipocytes displayed a drastic reduction in lipid droplet numbers compared to control untreated cells (Figure 2B). The number of Oil red O stained cells after a 24 h treatment with LE was decreased by up to 90% (*p* = 0.034) (Figure 2C). Quantification of the extent of staining by UV-vis spectroscopy showed a significant decrease (*p* < 0.001) in absorbance for LE-treated cells, compared to control cells (Figure 2D). This showed that LE does not affect the differentiation process but leads to decreased fat deposits in the cells as observed through the staining of the adipocytes. A study comparing effects of ACE inhibitors on PPARγ stimulation found that affinity to PPARγ was highest for telmisartan followed by lisinopril and valsartan at a concentration of 463, 2.9 and 6.2 µM, respectively [51]. It was thought that 1–10 μmol of telmisartan activates PPAR-γ but the concentration may be too small to exert an additional benefit on glucose metabolism [52]. The concentrations of LE used in the experiments may be insufficient to stimulate a change in PPARγ levels although a change in fat deposition was observed.

### 3.3. Stimulation of Lipolysis by Lemon Extract

**Figure 3:**
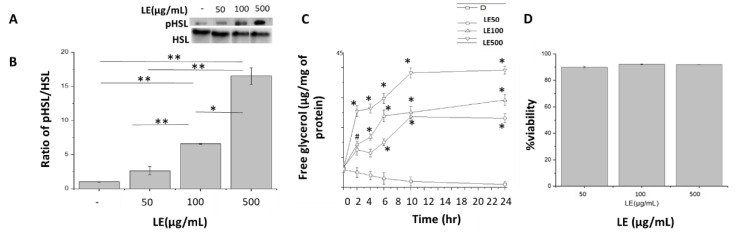
Stimulation of lipolysis by lemon extract (LE). (**A**) Blot for protein expression for pHSL and HSL in untreated differentiated cells, differentiated cells treated with LE dose of 50, 100 and 500 µg/mL. (**B**) Quantified protein expression for pHSL and HSL in untreated differentiated cells and differentiated cells treated with LE, at a dose of 50, 100 and 500 µg/mL. (**C**) Free glycerol release from treated and untreated cells after t = 0, 2, 4, 6, 10 and 24 h. * significant at *p* = 0.01 and # significant at *p* = 0.05. (**D**) Differentiated cells alone, differentiated cells treated with LE, at a dose of 50 µg/mL (LE50), differentiated cells treated with LE, at a dose of 100 µg/mL (LE100) and differentiated cells treated with LE, at a dose of 500 µg/mL (LE500) (**D**) Cell viability in the presence of LE, at a dose of 50, 100 and 500 µg/mL. ** significant at *p* = 0.01 and * significant at *p* = 0.05. For (**C**)

Hormone Sensitive Lipase (HSL) is a known marker for mobilization of lipids through lipolysis [35]. To understand the apparent decrease in lipid droplets, protein expression for pHSL was analysed. An increase in phosphorylation levels of HSL in response to increasing doses of LE was found. A 2.6, 6.5 and 16.6 fold change in pHSL levels in LE-treated differentiated cells was observed in comparison to control untreated differentiated cells (Figure 3A,B) after 10 h of treatment. To further test the hypothesis that the decrease in lipid droplet numbers was due to lipolysis, a free glycerol release assay was performed on mature adipocytes at t = 2, 4, 6, 10 and 24 h (Figure 3C) with different doses of LE. Hydrolysis of triglycerides through lipolysis lead to the release of free glycerol and free fatty acids (FFA). Free glycerol amounts of 23.08, 29.19 and 39.22 µg per mg of total protein was released after a 24-h treatment of 3T3-L1 cells with LE doses of 50, 100 and 500 µg/mL, respectively. At a LE dose of 500 µg/mL, a 4.02-fold higher release of free glycerol at t = 2 h was observed in comparison to t = 0 h (Figure 3C). A similar increase (~3.5–4.2 fold) was seen at t = 10 and t = 24 h for the 50 and 100 µg/mL LE doses. To confirm that LE did not lead to cell death, cell viability was assayed. The cells were treated with LE for 72 h and on an average 90.0 ± 0.5, 92.1 ± 0.2 and 91.9 ± 0.1% were viable even after 72 h (Figure 3D).

### 3.4. Lemon Extract Increases Insulin Sensitivity

**Figure 4:**
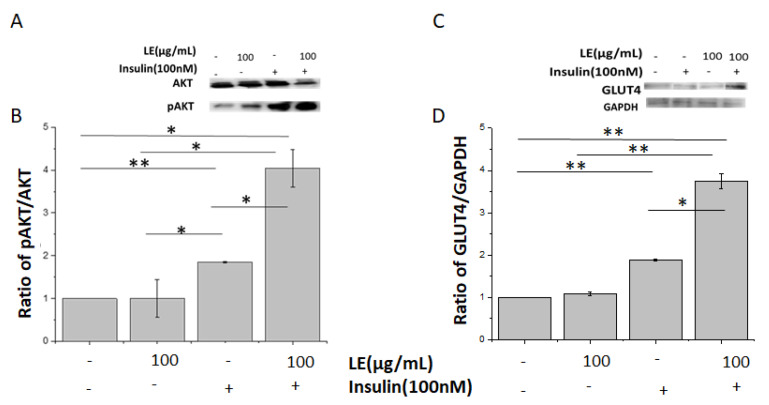
Lemon extract (LE) increases insulin sensitivity in differentiated adipocytes. (**A**) 3T3L1 differentiated adipocytes were incubated with or without LE for 10 h before being treated with 100 nM insulin for 10 min. Total AKT and phosphorylated AKT (pAKT) on Ser473 protein levels were assessed by immunoblotting in untreated differentiated cells (D), differentiated cells treated with LE (D+LE), differentiated cells treated with 100 nM insulin (D+I) and differentiated cells treated with LE and 100 nM insulin (D+LE+I). *n* = 3, *p* < 0.05. (**B**) Signal quantification of protein expression for AKT and pAKT in D (untreated differentiated cells), differentiated cells treated with LE (D+LE), differentiated cells treated with 100 nM insulin (D+I) and differentiated cells treated with LE and 100 nM insulin (D+LE+I). (**C**) 3T3L1 differentiated adipocytes were incubated with or without LE for 10 h before being treated with 100 nM insulin for 10 min. GLUT4 and GAPDH (loading control) were assessed by immunoblotting in untreated differentiated cells (D), differentiated cells treated with LE (D+LE), differentiated cells treated with 100 nM insulin (D+I) and differentiated cells treated with LE and 100 nM insulin (D+LE+I). *n* = 3, *p* < 0.05. (**D**) Quantified protein expression for GLUT4 and GADPH in untreated differentiated cells (D), differentiated cells treated with LE (D+LE), differentiated cells treated with 100 nM insulin (D+I) and differentiated cells treated with LE and 100 nM insulin (D+LE+I). ** significant at *p* = 0.01 and * significant at *p* = 0.05.

Protein Kinase B (AKT) is an important signaling molecule in the insulin pathway [53]. The insulin phosphorylation site Ser473 of AKT was more phosphorylated in control cells after insulin stimulation, with no effects on total AKT expression. Interestingly, LE incubation accentuated the insulin response, evidenced by a 3.74-fold increase in AKT Ser473 phosphorylation levels (compared to control; Figure 4A,B). A similar response was observed for GLUT4 expression, which was also increased in LE-treated cells (compared to untreated cells; Figure 4C,D).

### 3.5. Identification of Lemon Constituents by HPLC and HPLC/MS

**Figure 5:**
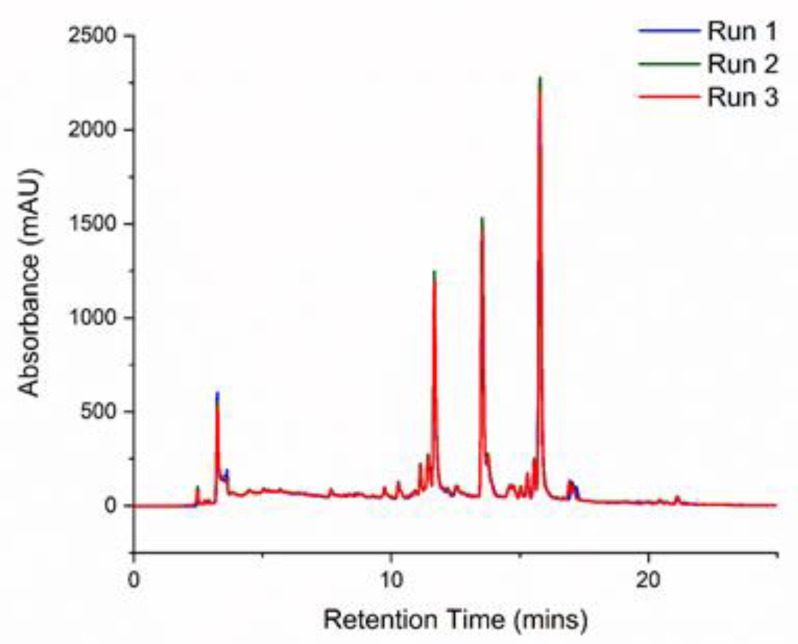
HPLC chromatogram of lemon extract. The extract was run on a C18 column with a mobile phase of water and acetonitrile; repeated thrice.

Having determined the effects of LE on adipocyte lipid utilization, further investigation was conducted to identify the compounds in the extract that could have been involved in the LE-induced increased lipolysis. HPLC-MS has been routinely used to identify active components in plant extracts [54,55]. HPLC measurements were first conducted to optimise the conditions for column separation of LE constituents. The chromatograms generated from these measurements are shown in Figure 5. The optimised conditions were run three times to ensure reproducibility of the spectrum. Then, high-resolution HPLC-MS was performed to obtain the molecular formulas of the different compounds in the extract. A total of 16 compounds were identified (see Table 2) through the mass/charge (*m/z*) ratios of their molecular ions ([M-H]^−^) and fragments [56]. This was compared to compounds in the PubChem database for identification. Citric acid was identified at a retention time (RT) of 1.3 min. Limonoids such as Nomilinic acid-17-Oglucoside, Nomilinic acid-4-Oglucoside and Limonin glucoside were found at RT = 13.3, 14.7 and 13.1 min, respectively. A mixture of flavonoids, Kaempferol acetyl dihexoside, Diosmetin-6, 8-di-C-hexoside, Diosmetin-7-O-rutinoside, Chrysoeriol 7-rutinoside, Chrysoeriol -7-O-neohesperidoside and Apigenin-6,8-di-C-glucoside, were also observed. A subfamily of flavonoids that contains a 3-hydroxyflavone backbone is referred to as flavonols. 6,8-C, CDiglucosyldiosmetin, Neodiosmin, Eriodictyol and Apigenin were the different flavonols present in our LE.

## 4. Discussion

The link between lemon juice dieting and insulin sensitivity has been established in humans previously: overweight women following a lemon detox diet (lemon juice with a mixture of maple and palm syrup) for 7 days showed reduced body fat and increased insulin sensitivity [15]. Here, we set out to understand the mechanisms underlying this finding. We demonstrate for the first time that LE treatment influences ACE gene expression levels in adipocytes. Specifically, 3T3-L1 adipocytes treated with LE decreased mRNA expression by ~80% after 10 h of exposure. We show that this process is accompanied by differentiation to mature adipocytes. This supports previous studies showing that higher ACE expression is related to increased adiposity and fat deposition in both, murine and human studies [57,58]. Increased production of ang II in adipose tissue has been observed in diet-induced obesity models [59]. The inhibitory effect of LE implies that it has the potential to act as an ACE inhibitor. Lemon and lime juice both inhibit ACE activity in a dose-dependent manner in mouse models [23]. 3T3-L1 cells also showed improved insulin sensitivity after treatment with LE, evidenced by increased p-AKT and GLUT4 levels. Several studies testing citrus fruits have found decreased insulin stimulated glucose uptake and improved glycemia and HbA1c levels [60,61].

Expression of GLUT4 and proteins involved in lipid breakdown showed coordinated upregulation in LE-treated adipocytes. In the LE-treated cells, there was an increase in free glycerol release along with perilipin and pHSL protein expression. It is well-established that these proteins and glycerol are a result of lipid breakdown [62,63]. Phosphorylation of HSL can also occur due to increased cAMP level activating a PKA pathway that is known to be involved in insulin signaling [64,65]. With these changes, it can be said that different compounds in our LE might be acting through PI3K/AKT pathway and could be used to treat obesity.

Mice with deletion of ACE (ACE-/-) have a reduced fat mass and improved glucose tolerance [66]. Diet-induced obesity in mice has been found to be reversed by ACE inhibitors [67]. Treatment with ACE inhibitor also decreases circulating leptin and insulin levels in rats [68]. A 12-week treatment of C57BL/6J mice, afflicted by diet-induced obesity, with an ACE inhibitor captopril increased peroxisome proliferator-activated receptor-g coactivator-1a, long-chain acyl-CoA dehydrogenase and HSL expression levels [67]. Another study analysing the long-term effects of ACE inhibitor enalapril in normotensive adult Wistar rats showed decreased ACE activity and enhanced the expression of adiponectin, HSL, fatty acid synthase and superoxide dismutase [69]. These findings support a potential link between ACE and HSL. Angiotensin inhibitors or ang II receptor inhibition augments natriuretic peptide (NP) levels [70]. Natriuretic peptides are a family of peptides that have diuretic, natriuretic, and vasodilator properties [71]. Atrial NP (ANP) induced lipolysis in human adipocytes is associated with a cGMP-dependent pathway that enhances HSL serine phosphorylation [72,73]. The guanylyl cyclase inhibitor LY-83583 inhibits ANP-mediated lipolysis and HSL phosphorylation in human adipocytes. Inhibition of ACE enzyme and ang II receptor improved ANP levels as well as cGMP levels in plasma and urine in a rat model of shunt-induced heart failure [74]. NPs have recently been shown to increase browning of adipocytes [75], which is usually associated with increased fat utilization and increased insulin sensitivity [76,77] and increased HSL phosphorylation [78]. The changes in pHSL levels seen in our study could be a result of increased NP levels or due to activation of PKA signaling involved in insulin sensitivity. Further studies are needed to identify association of LE and NP levels.

**Figure 6:**
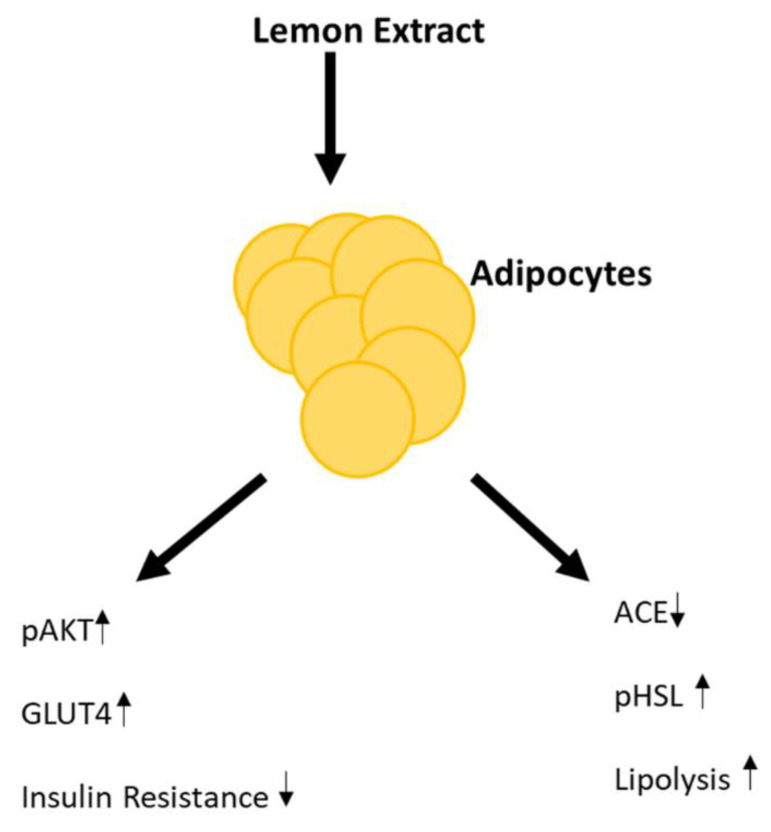
Proposed mechanism in which lemon extract (LE) induces insulin sensitivity and lipolysis in adipocytes. ↑- upregulation and ↓- downregulation.

Combining the different effects of LE, we here establish that LE alters the metabolism in mature adipocytes. Our study provides mechanistic support for the conclusion that LE’s effects on ACE expression and improved insulin sensitivity are related to each other. Moreover, LE acting as a potential ACE inhibitor could directly induce lipid breakdown (Figure 6). We have previously shown that ACE can act as a predictor for weight loss over a 24 h period [79]. Determining the effects of LE open doors for the use of lemon as an ACE regulator.

Similar to the experiments with rats, a study conducted with humans involving drinking lemon juice found decreased body fat, waist–hip ratio and high-sensitive C-reactive protein (hsCRP) in serum [15]. Similar results were obtained with other citrus fruits such as orange juice, bergamot extract and grapefruit, which all have beneficial effects on lipid variables. Drinking orange juice every day lowered concentrations of LDL and the LDL/HDL ratio [22]. Another study involving supplementation of every meal with grapefruit decreased body weight [21].

To begin narrowing down what are possible ingredients in LE responsible for the effects observed in this study, we conducted HPLC/MS analysis of the LE we prepared. We were able to identify 16 different compounds. A few of these or closely related compounds have been studied with respect to their biological activities. Rat plasma triacylglycerol level reduced after oral administration of mixture of Chrysoeriol-7-O-neohesperidoside and apigenin prepared from the leaves of *Salix matsudana* [80]. Mouse fed with 0.15% Kaempferol, prepared from unripe soybean leaves, had a reduced body weight, blood glucose due to down regulation of PPARγ and sterol regulatory element-binding protein expression in liver [81]. Kaempferol is thought to improve glucose uptake in rats through PI3K/AKT pathway [82]. In our study, we found increase in insulin sensitivity through AKT pathway showing that Kaempferol acetyl dihexoside and Kaempferol-Osophoroside -Oglucoside could be the active compounds in our extracts.

The commonly studied flavonoid in citrus fruits studies are naringin (Flavonoid-7-o-glycosides), hesperidin (flavonoid glycoside) and nobiletin [60]. Naringin and nobiletin were not identified in our extract through MS technique. Different diosmetin species (such as Diosmetin-6, 8-di-C-hexoside, 6,8-c-c diglucosyldiosmetin isomer, c-c diglucosyldiosmetin, diosmetin 7-O-neohesperidoside and diosmetin-7-O-rutinoside) found in our extract are derived from hydrogenation of hesperidin. These molecules inhibit the accumulation of triglycerides, increase HDL and decrease VLDL-TG sections [83,84]. Naringenin has anti-inflammatory properties and promotes insulin sensitivity [83,85]. Mouse model studies found that supplementation of a high-fat, cholesterol-containing diet with naringenin attenuated weight gain and adiposity, enhanced insulin signaling, lowered plasma lipids and prevented systemic inflammation [86]. It also reduced adipose tissue mass [86]. Hesperidin decreases plasma and hepatic cholesterol and triacylglycerol by inhibiting the hepatic enzymes [84,87]. Type-2 diabetic animals supplemented with hesperidin improved hyperlipidemia and hyperglycemia and enhanced hepatic and adipocyte PPARγ protein expression [88]. Healthy volunteers consuming hesperidin for 4 weeks showed a reduction in cytokines and inflammatory markers in circulation [89,90,91]. Limonoids are highly oxygenated triterpenoid compounds present in citrus species and are responsible for bitterness in citrus juices [92]. The major types of limonoids are limonin and nomilin. We observed a mixture of limonoids in our extract (see Table 1). Male C57BL/6J mice fed a high-fat diet (HFD) supplemented with 0.2% *w/w* nomilin for 77 days had lower body weight, serum glucose and insulin, and enhanced glucose tolerance [93]. These changes were associated with TGR5 signaling and nomilin has been identified as a novel agonist for TGR5 [93]. TGR5 is a novel G protein-coupled receptor that promotes the elevation of intracellular cAMP levels in adipose tissue [94]. Eriodictyol supplementation in mice fed with a high-fat diet for 16 weeks improved insulin resistance by suppressing hepatic gluconeogenesis, enhancing glucose utilization, and modulating the production and release of gastric inhibitory polypeptide and glucagon-like peptide-1 [95]. It also significantly downregulated the expression of lipogenesis-related genes. Thus, it is likely that several of the individual compounds in LE might work in synergy to create the beneficial effects seen in our study.

## 5. Conclusions

The LE-dependent fat breakdown properties observed in mature adipocytes rationalizes its use as dietary intervention. The link between LE and ACE expression levels and LE as an ACE inhibitor also provides a mechanism based upon which LE is not only useful in the management of obesity but could also be beneficial in the management of cardiovascular diseases.

## Figures and Tables

**Table 1:** HPLC gradient program.

Time (min)	A: Water	B: Acetonitrile	Flow Rate (mL/min)
0	95	5	1
10	75	25	1
20	60	40	1
30	50	50	1
35	5	95	1
40	5	95	1
50	95	5	1
60	95	5	1

**Table 2:** List of compounds found in lemon extract using HPLC/MS.

RT (min)	[M-H]-	Mol. Formula	MS Fragments (m/z)	Name	Class
1.3	191.02	C_6_H_7_O_7_	133, 111	(iso)citric acid	Organic acid
5.9	771.201	C_33_H_39_O_21_	695, 547, 415, 375, 353, 285, 191	Kaempferol-Osophoroside-Oglucoside	Flavonol acylated glycoside
13.1	711.28	C_34_H_47_O_16_	693, 549, 341	Nomilinic acid-17-O-glucoside	Limonoid
14.7	693.2775	C_34_H_45_O_15_	531, 443, 341	Nomilinic acid-4-Oglucoside	Limonoid
2.6	651.	C_29_H_31_O_17_	507, 417, 341	Kaempferol acetyl dihexoside	Flavonoid-3-o-glycosides
13.1	649.2513	C_32_H_41_O_14_	413, 341	Limonin glucoside	Limonoid
8.9	625.17	C_28_H_33_O_16_	383, 312	Diosmetin-6, 8-di-C-hexoside (Lucenin-2,4′-methyl ether)	flavonoid-7-o-glycosides
2.3	623.1622	C_28_H_31_O_16_	605, 533, 503, 579, 443	6,8-C, CDiglucosyldiosmetin isomer	C-flavone glycoside
2.7	623.1565	C_28_H_31_O_16_	605, 533, 503, 413, 329	C,CDiglucosyldiosmetin	C-Flavone glycoside
14.9	609.1825	C_28_H_33_O_15_	301.07	Diosmetin-7-O-rutinoside (diosmin)	Flavonoid-7-o-glycosides
15.7	609.1824	C_28_H_33_O_15_		Chrysoeriol 7-rutinoside	flavonoid-7-o-glycosides
13.6	607.1668	C_25_H_31_O_15_	299,284	Diosmetin 7-O-neohesperidoside Neodiosmin	Flavone
14.7	607.1668	C_25_H_31_O_15_	299,283.8	Chrysoeriol-7-O-neohesperidoside	Flavonoid-7-o-glycosides
13.3	595.1668	C_27_H_31_O_15_	505, 457, 427, 421, 409, 391, 379, 355, 337, 325, 307, 295	Apigenin-6,8-di-C-glucoside	Flavonoid
13.0	287.0556	C_15_H_11_O_6_	151,135,125,107	(2S)-Eriodictyol	Flavone
36.7	269.24	C_17_H_37_O_2_	225,201,151	Apigenin	Flavone

## Supplementary Materials

The following are available online at https://www.mdpi.com/2072-6643/12/8/2348/s1, Figure S1: Western blot of ACE and GADPH, Table S1: Quantification of protein expression using Image J software.
