# Supplementary material for: Lemon Extract Reduces Angiotensin Converting Enzyme (ACE) Expression and Activity and Increases Insulin Sensitivity and Lipolysis in Mouse Adipocytes
Source: Nutrients. 2020 Aug 6;12(8):2348. doi: 10.3390/nu12082348 (PMC7468735; doi:10.3390/nu12082348)
Supplement: Supplementary file 1 [file nutrients-12-02348-s001.pdf]

nutrients

MDPI

Lemon Extract Dose (µg/ml)	Run 1	Run 2	Run 3	Average fold change	SD
0	0.987449	1	0.9897	0.992383	0.006692
50	0.489014	0.438364	0.423906	0.450428	0.034189
100	0.482344	0.520875	0.454776	0.485998	0.0332
500	0.641399	0.639431	0.538766	0.606532	0.058695

Table S1: Quantification of protein expression using Image J software.

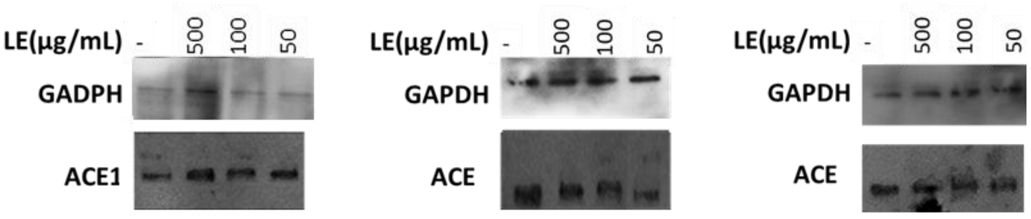

Figure S1: Western blot of ACE and GADPH. LE: Lemon Extract
